# A Qualitative Exploration of COVID+ Learning Network Webinars: Healthcare Learnings at Pace in a State of Rapid Change

**DOI:** 10.1177/21501319241295672

**Published:** 2024-11-11

**Authors:** Brett Morris, Jo-Anne Rayner, Chantelle Bartlett, Deirdre Fetherstonhaugh, Emmalee McArdle, Amber O’Brien

**Affiliations:** 1 Safer Care Victoria, Melbourne, VIC, Australia; 2 Latrobe University, Bundoora, VIC, Australia

**Keywords:** learning networks, health services, clinicians, primary care, community healthcare, COVID-19 pandemic

## Abstract

**Background:**

Learning Networks are increasingly used to educate clinicians and disseminate information to health professionals. During the height of the second COVID-19 lockdown in Victoria, Australia, COVID+ Learning Network webinars were introduced as a mechanism for disseminating emerging evidence and up-to-date information to health service managers, and primary care and community healthcare clinicians, and for obtaining feedback from the healthcare sector.

**Methods:**

A qualitative descriptive study design was used to explore the COVID+ Learning Network webinars from users’ perspectives. Fifteen webinar participants from different professional backgrounds, roles during the COVID-19 pandemic, and geographic locations were individually interviewed.

**Results:**

The webinars attracted state-wide engagement and participants described them as an effective way to inform and support health services, and primary care and community healthcare clinicians. However, data analysis revealed important considerations for using Learning Networks to disseminate information in the future. In particular, the importance of tailoring the webinars to address the specific needs of the different participant groups was highlighted.

**Conclusion:**

Health service managers, and primary care and community healthcare clinicians, require information pertinent to their specific roles, with consideration of geographic and socio-economic factors important to planning webinar content. Future learning network webinars would benefit from a more targeted approach to address the specific informational needs of the groups participating.

## Introduction

The COVID-19 pandemic presented a major challenge for the planning and delivery of care across healthcare systems in Australia and internationally.^1^ The Australian healthcare system includes universal access to health and medical care (public hospital and medical service provision) through Medicare,^2^ as well as private medical (primary care) and hospital service provision. The Commonwealth government is responsible for funding primary care and aged care services and State and Territory governments are responsible for public health service delivery including community healthcare. Health services in Victoria, where this research was undertaken, operate through a model of devolved governance, where Boards, are responsible for overseeing healthcare delivery.^3^

**Figure 1.**
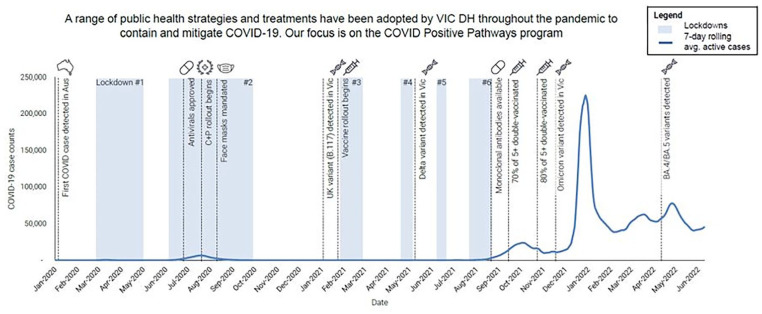
Victorian Department of Health COVID-19 response timeline/strategies^a^. ^a^Included with the permission of Victorian Department of Health.

The state of Victoria experienced the greatest COVID-19 pandemic disease burden in Australia,^4^ including 6 lockdowns, necessitating state-wide public health control measures (see Figure 1).

Responding to increasing cases of COVID-19 during Victoria’s second wave, out-of-hospital monitoring was implemented through the establishment of a COVID+ Pathway in August 2020.^5^ The COVID+ Pathway was a collaborative model of care involving the Victorian public health unit, hospital services, primary care, community healthcare, and the primary health network (PHNs), to support people with COVID-19 to isolate at home with appropriate care and monitoring.^5^ Victoria has 6 PHNs that: assess community healthcare needs and direct health services to meet those needs; support health services to connect to improve care; and strengthen primary care through the use of health pathways (online referral pathways and resources) to assist clinicians.^6^

The rapid and continuous transfer of a centralized source of evidence-based information can be slow in diverse and geographically disparate health services. This was further emphasized during the COVID-19 pandemic with the initiation of public health measures such as lockdowns. To overcome this, and as part of the COVID+ Pathway initiative, an online COVID+ Learning Network (the Learning Network) was developed. This Learning Network, based on the Learning Health System model, which merges healthcare delivery with research, data science, and quality improvement processes,^7^ was a collaboration between Safer Care Victoria (SCV) and the Victorian Department of Health (DoH). An executive steering group, including senior clinical leads, executive members, and DoH representatives, was established to oversee the Learning Network, which aimed to facilitate knowledge and resource sharing between health services to deliver the COVID+ Pathway. The Learning Network comprised weekly COVID-19 webinars, a secure portal for shared COVID-19 clinical resources; and a SCV Clinical Conversations series. The webinars were a forum to support health services managers and clinicians in primary care and community healthcare; and a centralized source for communication on policy changes, emerging evidence, and guidance from the National Clinical Evidence Taskforce COVID-19.^8^

The webinars were promoted using a Learning Network email distribution list of over 1,000 people drawn from existing DoH and SCV stakeholder lists, and in the weekly bulletin sent to all Victorian Health Service chief executive officers. Email recipients were encouraged to circulate the webinar invitation within their networks and webinar attendees could register to receive direct notifications of future sessions via a dedicated Learning Network email address. As this email distribution list expanded, attendees were required to register for each webinar from April 2022.

Emerging COVID-19 issues and evidence, policy changes, health service managers, and primary care and community healthcare clinicians’ needs and experiences, drove the webinar topics. During each webinar, attendees were asked to email suggestions for future webinar topics. All webinars were recorded and available on the SCV website. Between September 2021 (Learning Network launch) and August 2022 (commencement of research), 23 webinars were aired. Initially aired weekly, the frequency of the webinars was reduced to monthly to align with the diminished demand for COVID-19 information.

Overall, the webinars attracted a sizeable number of attendees (n = 7787), either live (n = 3630) or later viewing of the recorded webinar (n = 4157). The median number of live and recorded webinar attendees per webinar was 158 (range = 84-294) and 181 (range = 30-975) respectively. Live webinar attendance was highest for Webinar 13 and the recording of Webinar 19 attracted the most views (Table 1).

**Table 1.** Webinar Dates, Topics, and Attendance.

No.	Year/date 2021	Topic	Attendees	
			Live	Recording
1	29 September	Experiences and learnings in delivering the COVID+ pathway—The Northwest Melbourne experience	196	313
2	6 October	Innovations in COVID-19 care delivery—Northern Health	174	207
3	13 October	COVID+ pathways experience and learnings from the ED—The Royal Melbourne Hospital	137	201
4	20 October	Pediatric COVID+ pathways in practice	145	127
5	27 October	Launch—updated COVID-19 care pathways	159	119
6	3 November	COVID+ pathways experience—Barwon health	119	139
7	10 November	Bendigo health’s approach to COVID+ pathways	159	138
8	17 November	Improvement science at pace—standardization in the medium-risk COVID+ pathway	139	145
9	24 November	Maternity care—COVID-19+ pathway and streaming models	159	125
10	1 December	COVID navigators and supporting discharge	139	171
11	8 December	Care after the wave: how to prepare for the patient with Long COVID	210	264
12	15 December	Expediting discharge—timely care diagnostics to identify opportunity areas	139	145
	2022			
13	19 January	COVID+ pathways—critical updates and discussion	294	172
14	2 February	NHS London region—their experience of managing omicron—critical care and antivirals	238	160
15	16 February	NSW experience and learnings from Omicron	92	111
16	2 March	North-western Melbourne Primary Health Network	89	112
17	16 March	NHS London region—GP + respiratory	164	84
18	30 March	COVID+ pathway for aboriginal and/or Torres strait islanders	159	53
19	27 April	The big reset: COVID as transformation	258	975
20	25 May	Long covid—local VIC	118	212
21	29 June	GP RCs, rural GP clinics, early therapies access	84	72
22	27 July	Supporting community access to COVID therapies	157	82
23	24 August	Update from the wellbeing for healthcare workers initiative	102	30

Findings of exploration of the Learning Network webinars as a mechanism for disseminating emerging evidence and up-to-date information to health service managers, primary care, and community healthcare clinicians are presented.

## Methods

### Design and Sample

The project used a qualitative design, with information sought directly from those who attended the webinars,^9,10^ to explore their experiences of the Learning Network. Purposive sampling,^11^ was used to select participants from the webinar attendee lists. Webinar registration details (gender, role, location of employer, and number of webinars attended) were used to develop a recruitment matrix, so a diverse group of participants could be interviewed to explore issues from different perspectives. Emails were sent to attendees who had provided contact details inviting them to participate in a single semi-structured interview. Using the matrix the first attendees who agreed were recruited. To ensure maximum variation from hard-to-recruit groups,^12^ direct emails were sent to 18 medical specialists, 24 primary-care clinicians, and 42 pharmacists who attended the webinars. None of the research team was involved in the recruitment of participants.

### Data Collection

All authors were involved in interviewing participants. As some of the project team members were early career researchers, some interviews were conducted with the guidance of a team member with more qualitative research experience. A semi-structured interview guide was developed and piloted with colleagues. In line with the qualitative descriptive design,^9,10^ the interviews aimed to explore participants’ experiences of the webinars, including the number they attended, and what they gained from attending. Informed consent was obtained from each participant to record the interview. The interviews were conducted virtually between August and October 2022, via the Microsoft Teams^13^ platform, audio-recorded, and transcribed using the same application. All identifying information was deleted to maintain participant anonymity.

### Data Analysis

The interviews were analyzed using reflexive thematic analysis.^14,15^ The data analysis focused on the acceptability of the webinars as a source for disseminating emerging evidence to health services and clinicians. A core feature of qualitative analysis is reflexivity, that is, there is no ultimate truth, only perspective and truthfulness—reflection on how the project design, collection, and interpretation of data shape the findings. Reflexive qualitative data analysis does not set out to discover the “truth,” rather the final analysis is the product of deep and prolonged data immersion, thoughtfulness, and reflection, something that is active and generative^15^ (p. 591).

Four of the researchers were involved in coding the transcripts to eliminate bias and enhance the trustworthiness of the qualitative data analysis. Creating trustworthiness in the data analysis involved 5 stages:^16^ compiling (transcribing, reading, and familiarizing); disassembling (creating codes); reassembling (mapping codes to create themes); interpreting (making analytical conclusions); and concluding (interpreting themes in line with the research question).

The transcripts were divided between the 4 researchers who coded them separately. Each transcript and the codes were discussed in weekly meetings, to iteratively compare the coded data and improve the rigor of analysis until a consensus was reached. Consensus required agreement by 3 of the 4 coders with the final codes being representative of all 4 researchers’ perspectives.

Methodological rigor was maintained by using the Consolidated Criteria for Reporting Qualitative Data^17^; sampling of participants, data collection, and analysis^18^; independent analysis before coding consensus, and refinement of themes.^19^ Direct quotes are used to illustrate the findings, including a reference to the number of webinars attended (see Table 2) the exact number of which many participants could not remember.

**Table 2.** Participants (n = 15).

ID	Role	Role during 2021-22 COVID-19 pandemic	Health service location	Webinars attended
ID01	Director of Clinical Services	Organized the Health Service COVID-19 response	Rural	“7”
ID02	Director of Clinical Services	Led the Health Service COVID-19 response	Rural	“ 5 to 6”
ID05	Community Healthcare Manager	Led COVID-19 Pathway in community healthcare service	Outer Metropolitan	“8 to 9”
ID06	Chief Executive Officer	Oversight of COVID-19 model of care	Rural	“All”
ID07	Director of Integrated Community Healthcare	Oversight of COVID-19 community healthcare services	Outer Metropolitan	“Most”
ID08	Executive Director of Health Partnership	Organized the PHN COVID-19 response	Inner Metropolitan	“Most
ID09	Executive Director of Clinical Services	Organized the Health Service COVID-19 response	Inner Metropolitan	Most”
ID11	General Manager Primary Care	Managed the Primary Care COVID-19 response	Outer Metropolitan	“Minimal”
ID12	Chief Executive Officer	Managed the Health Service COVID-19 response	Rural	“Most”
ID13	Chief Executive Officer	Managed the Health Service COVID-19 response	Rural	“On and Off”
ID15	Divisional Director of Medicine	Managed the Heath Service COVID-19 response	Inner Metropolitan	Managing remember”
ID16	Geriatrician	Clinical-led of SCV Executive Steering Committee and managed Statewide COVID-19 response	Inner Metropolitan	“According to the topic”
ID17	Academic/Clinical Advisor PHN/Primary Care Clinicians	Led the Health Service COVID-19 response project and worked as a primary care clinician managing COVID+ patients	Regional	“Minimal”
ID18	Senior Allied Health Practitioner	Senior Allied Health Practitioner in Health Service	Inner Metropolitan	“1”
ID19	Community Healthcare Nurse Unit Manager	Led the Health Service COVID-19 Pathway virtual home monitoring	Regional	“10”

Abbreviation: PHN, Primary health network (In Victoria PHNs (n = 6) assess community healthcare needs and direct health services to meet those needs; support health services to connect to improve care; and use health pathways (online referral pathways and resources to assist primary care and community healthcare clinicians.

### Ethical Considerations

The research was developed using the Australian National Health and Medical Research Council Guidelines and approved by the La Trobe University Human Research Ethics Committee [Ethical Approval No. HEC22112].

## Results

Twenty participants initially agreed to be interviewed, however, 5 were unavailable when contacted. The 15 participants interviewed were diverse in terms of their geographic location, gender (9 women and 6 men), and role (see Table 2). Their backgrounds included medicine, nursing, and allied health, with roles in varied levels of healthcare leadership/management, clinical care in acute, subacute, primary, and community healthcare contexts, and academia.

The Learning Network webinars attracted state-wide engagement from health service managers, and primary care and community healthcare clinicians. Participants described it as an effective way to rapidly inform, educate, and support, although the number of webinars they attended varied. Some described selecting the webinars according to the topic being of interest or having relevance to their role. The webinars were described as *interesting*, *relevant*, and *informative* for planning management of COVID-19 outbreaks, especially in rural and regional health services. One participant stated the webinars were “*a good way to engage different groups in a shared problem*.” In addition, many participants stated that they disseminated the webinar information to colleagues and that the information increased their ability to effectively communicate with other clinicians. Others were grateful for the broader view of COVID-19 management and for gaining an understanding of how other health services were managing outbreaks.

*Some of the things I got to take back to my leadership team were some of the pathways out of COVID and what to look forward. Those sorts of things I found valuable for our organisation (ID.02).*

Participants favored the webinar’s scheduled time (start of the workday), and length (an hour), and that webinar recordings were accessible if they were unable to attend live.

*The 8:00 a.m. is perfect because you can either listen while travelling or at work while doing emails or whatever (ID.12).*

*I think the concept of the Learning Network, the regularity of the webinars, and the time of day just worked super well (ID.05).*

However, the analysis revealed one over-arching theme: 1. *Usefulness and Engagement* with 4 sub-themes: 1.1.*Specific information needs*; 1.2. *Diverse information needs*; 1.3 *Devolved governance*; and 1.4 *Considerations for future webinars*.

### Usefulness and Engagement

Participants wanted webinars relevant to their role, particularly their role during the COVID-19 pandemic, with many suggesting the need for targeted webinars. However, this feedback was often conflicting. Some reported that the webinars were too clinically focused with the complexity of information provided beyond their comprehension. Conversely, others argued that the webinar content was not targeted to clinicians, with a particular gap highlighted for primary care clinicians who were providing frontline COVID+ care. Others reported that the webinars were too policy-orientated. The webinars were described by 1 participant as being like *town hall meetings*, where important, up-to-date generalist information was provided which may or may not have been relevant to all attendees.

#### Specific information needs

The suggestion that the webinars should have had more content related to individual roles during the COVID-19 pandemic came from across the participant spectrum. Those in administration or managerial roles felt that the webinars lacked useful health system data on managing the response from a health service perspective, while clinicians wanted information pitched to address the day-to-day care they were delivering.

*Sharing practice across the metropolitan health service networks has been valuable, however, there was a lack of useful data on how to manage the pandemic response from a health service perspective (ID.08).*

*Your network was not designed with primary care clinicians in mind. . .Primary health network leaders would attend and find it useful, but clinicians would find it a bit high-level (ID.17).*

*Service delivery, pathway or model development, and interacting with primary care, interested me. All the clinical stuff that’s not me (ID.05).*

Rural participants specifically reported that the information presented in the webinars was *metrocentric*; and that there was *a need for a rural voice* as rural and remote health services are *always expected to adapt* to what is done in metropolitan health services. These participants reported that the webinars did not account for the differences in health service types or models of care; and did not address the limited access rural services have to clinical services, resources, and the COVID-19 response model. Another important consideration raised by regional and rural participants was limited internet access, which was a barrier to attending the webinars, and severely disenfranchised some communities.

*It needs to be neutral not metro-centric, not rural, and regional-centric. It needs to cover all. Perhaps we can have separate webinars, one for Metro and one for regional (ID.01).*

*You were trying to cater to a broad audience, so it can make meetings quite unwieldy, and 90% switched off. I think awareness of that small rural [health services] have aged care beds and have [different] directives coming from aged care. It was not necessarily recognising the challenges that existed at a local level (ID.13).*

#### Diverse information needs

Many participants reported that they would have liked the webinars to have more information on the needs of culturally and linguistically diverse (CALD) and low socio-economic groups in their community that were over-represented among people with COVID-19.

*We saw that people from multicultural and disadvantaged communities were disproportionately impacted by COVID-19, in rates of hospitalizations, ICU admissions, deaths, and low vaccination rates. Lots of complexity. This was great information, but we must tailor it significantly to work with our populations or people experiencing homelessness (ID.07).*

*You must account for the social determinants of health and the impact of socioeconomic status. Poor people, vulnerable people (ID.15).*

#### Devolved governance

Finally, whilst the COVID-19 pandemic necessitated a rapid centralized approach, some participants were uncertain if Victoria’s approach to devolved health service governance presented a challenge to using Learning Networks for long-term information dissemination and engagement. Some participants reported that health services do not generally share information, and the webinars facilitated this sharing, while others remained sceptical.

*Victoria’s decentralized model has its strengths and weaknesses, so I think sharing practices across health service networks, has been valuable (ID.08).*

*You’re going to have a transparent health service that’s prepared to comfortably share its intellectual property [IP] through a webinar (ID.11).*

The devolved governance model has previously been associated with reduced sharing of ideas and collaboration between health services and primary care.^3^ Despite this limitation, participants agreed that the Learning Network webinars provided an opportunity to do things differently and to connect to colleagues across the geographic and health service divides.

#### Future learning networks

All participants suggested many common topics that they would like to see included in future Learning Network webinars. The most frequently reported topics, in order of importance, were the management of chronic diseases, in particular, primary care management of cardiac disease and diabetes; immunization; urgent care in rural and remote areas; community healthcare and home care; and workforce issues, particularly the medical workforce shortages in rural and remote health services.

*There’s huge scope [for the use of Learning Networks] in chronic disease management (ID.18).*

*Urgent care in rural areas and managing medical workforce shortages (ID.06).*

*Staffing, how to navigate with limited pharmacy resources, and limited medical resources (ID.01).*

Participants from regional and rural health services particularly favored the Learning Network webinars because they reduced professional isolation, enabling them to keep up to date with information usually restricted by geographic isolation. A small number of participants however reported that they missed face-to-face sharing meetings to share ideas and experiences as “*webinars do not give you that opportunity to make that connection one-on-one*.”

*It was a great resource, especially for small organizations where they have no or few educators. This is a wonderful way of educating staff. I hope this does not end with COVID-19 ending (ID.01).*

## Discussion

Learning Networks webinars seem to be an acceptable mechanism for effectively distributing emerging information in a rapid and widespread manner. Responding to the COVID-19 pandemic, and the rapidly changing needs of health service provision, SCV initiated, and provided, an innovative Learning Network to virtually disseminate up-to-date information on the care of people diagnosed with COVID-19 to health services managers, and primary care and community healthcare clinicians. This was achieved in an exceptionally short timeframe, with input and advice from local, national, and international experts. Participants reported that the webinars were a means of swiftly communicating and collaborating with those responsible for health service provision and clinical care. A qualitative exploration of an international Learning Health Network initiative established during the COVID-19 pandemic also generated positive reports from users.^20^

Internationally the use of virtual technology to rapidly educate and inform health service personnel is well documented^21-30^; during the COVID-19 pandemic to support online teaching and learning,^21,22^ and to support health services and primary care clinicians respond to the pandemic.^21,22,25,26^ Learning Networks have also been used in many countries to provide clinicians with the most up-to-date evidence^27-30^; and to facilitate system-level improvement through the rapid provision of data, evidence, and experience.^28-31^

The acceptability of Learning Network webinars was ultimately judged by users,^32^ especially primary care clinicians,^22,33^ rural and remote health services, and clinicians providing care to CALD^34^ and socio-economically deprived communities during the COVID-19 pandemic. The timing, length, and frequency of Learning Network webinars were well received by all; however, the topic choices seem to have been reactive and failed to address the needs of all groups attending. Considering the urgency for and speed of information dissemination during a global pandemic, this is understandable.

Australia has a large land mass with a smaller population (nearly 26 million) compared to other developed countries,^35^ and a third of Australia’s population live in regional and rural locations. They have limited access to health care services and poorer health outcomes compared to their metropolitan counterparts,^36^ as ongoing health service provision is challenged by small populations, limited access to resources, and the tyranny of distance. For many regional and rural populations, primary care clinicians, became the main source of information about COVID-19,^37^ so their specific information needs cannot be minimized. Similarly, the information needs of CALD,^34^ and other at-risk groups were highlighted as inadequate among participants. No webinars addressed the needs of CALD and socio-economically deprived groups and only one addressed the COVID-19 care needs of Aboriginal and/or Torres Strait Islanders, despite Victoria having the most culturally and linguistically diverse population of all Australian States and territories.^38^

Notwithstanding these shortcomings, the coordination of regular, timely, and accurate information via virtual systems such as the Learning Network, has allowed for clear consistent messages and the development of “trusted sources of truth”^39^ (p. 734). The COVID-19 pandemic has accentuated the need for change, as the “old way” of doing things is gone^40^ (p. 2). Learning Networks are part of this change,^20^ facilitating faster information-sharing and knowledge dissemination across multiple sites and to numerous people, functioning as “flows of information, . . .resources,. . .and support”^41^ (p. 4). Nevertheless, the findings suggest that conducting a needs assessment of the target group(s) should be considered as part of future Learning Network webinars.^42^

### Limitations

These research findings are limited by the research design and sampling bias. The study sought to explore rather than quantitatively evaluate the impact of the Learning Network webinars, and as such the results are not generalizable. The inclusion of additional participants may have yielded further findings. However, research rigor does not depend on the sample size but on the scope and purpose of the project^43^ and the ability of the participants to provide the data needed for analysis.^44^ Secondly, the sample included a small number of self-selected participants. Invitations to participate were weighted toward individuals who were at the high and low extremes of engagement with the Learning Network, however, the perspectives of healthcare staff who did not engage are not represented. Finally, it would have been beneficial to have included the experiences of consumers involved in the Learning Network webinars. Despite the limitations, the research design, particularly the sample, the selection of participants, and the data analysis contributed to the trustworthiness of the findings.^19^

## Conclusion

The findings suggest there was strong engagement by and support for, the COVID-19 Learning Network webinars among health service managers, and primary care and community healthcare clinicians. The Learning Network was seen as a valuable platform for learning, collaboration, engagement, and accessing resources, with recommendations for extension to additional content areas to address shared healthcare concerns. However, to achieve their desired effect and engage groups more fully, future Learning Network planning must incorporate needs analyses of the type of information participants require, and/or consider different webinar series created for, and targeted to, the varied learning needs of different participant groups.
